# Metabolic Profiling of Retrograde Pathway Transcription Factors Rtg1 and Rtg3 Knockout Yeast

**DOI:** 10.3390/metabo4030580

**Published:** 2014-07-08

**Authors:** Zanariah Hashim, Yukio Mukai, Takeshi Bamba, Eiichiro Fukusaki

**Affiliations:** 1 Department of Biotechnology, Graduate School of Engineering, Osaka University, 2-1 Yamadaoka, Suita, Osaka 565-0871, Japan; E-Mails: hashim_zanariah@bio.eng.osaka-u.ac.jp (Z.H.); takeshi_bamba@bio.eng.osaka-u.ac.jp (T.B.); 2 Department of Bioscience, Nagahama Institute of Bio-Science and Technology, 1266 Tamura, Nagahama, Shiga 526-0829, Japan; E-Mail: y_mukai@nagahama-i-bio.ac.jp

**Keywords:** metabolomics, retrograde (RTG) pathway, bHLH proteins, yeast *Saccharomyces cerevisiae*

## Abstract

Rtg1 and Rtg3 are two basic helix-loop-helix (bHLH) transcription factors found in yeast *Saccharomyces cerevisiae* that are involved in the regulation of the mitochondrial retrograde (RTG) pathway. Under RTG response, anaplerotic synthesis of citrate is activated, consequently maintaining the supply of important precursors necessary for amino acid and nucleotide synthesis. Although the roles of Rtg1 and Rtg3 in TCA and glyoxylate cycles have been extensively reported, the investigation of other metabolic pathways has been lacking. Characteristic dimer formation in bHLH proteins, which allows for combinatorial gene expression, and the link between RTG and other regulatory pathways suggest more complex metabolic signaling involved in Rtg1/Rtg3 regulation. In this study, using a metabolomics approach, we examined metabolic alteration following *RTG1* and *RTG3* deletion. We found that apart from TCA and glyoxylate cycles, which have been previously reported, polyamine biosynthesis and other amino acid metabolism were significantly altered in RTG-deficient strains. We revealed that metabolic alterations occurred at various metabolic sites and that these changes relate to different growth phases, but the difference can be detected even at the mid-exponential phase, when mitochondrial function is repressed. Moreover, the effect of metabolic rearrangements can be seen through the chronological lifespan (CLS) measurement, where we confirmed the role of the RTG pathway in extending the yeast lifespan. Through a comprehensive metabolic profiling, we were able to explore metabolic phenotypes previously unidentified by other means and illustrate the possible correlations of Rtg1 and Rtg3 in different pathways.

## 1. Introduction

Rtg1 and Rtg3 are two basic helix-loop-helix (bHLH) transcription factors found in yeast *Saccharomyces cerevisiae* and are known regulators of the mitochondrial retrograde (RTG) response [1]. The mitochondrial RTG response is the signaling pathway from mitochondria to the nucleus triggered by the functional states of mitochondria [2,3,4]. This pathway maintains a continuous supply of 2-oxoglutarate, a precursor of glutamate and glutamine biosynthesis, by activating the anaplerotic metabolism of citrate and oxaloacetate via the glyoxylate cycle when respiratory metabolism through the tricarboxylic acid (TCA) cycle is compromised in the event of mitochondrial dysfunctions. It is thus one of the important regulations that ensure a continuous supply of precursors for biosynthetic reactions through alternative metabolic pathways.

Rtg1 and Rtg3 form heterodimers and translocate from the cytoplasm to the nucleus when RTG response is activated [5]. This translocation depends on the phosphorylation state of Rtg3, and the transcriptional activation domain is contained within Rtg3 [6]. Rtg1/Rtg3 complex binds to R-box (GTCAC), which differs from the canonical E-box site (CANNTG) to which most other bHLH proteins bind [1]. Among Rtg1/Rtg3 target genes are several TCA cycle genes, but the prototypical target is *CIT2* [4], which encodes a peroxisomal citrate synthase in *S. cerevisiae*. The transcript level of *CIT2* was reported to be elevated in petite cells (cells that contain nonfunctional, mutated mitochondrial DNA or have completely lost their mitochondrial DNA), demonstrating the activation of the RTG response under dysfunctional mitochondria. Although there is a large difference in *CIT2* expression between ρ^+^ and ρ° cells, Rtg1 and Rtg3 appear to interact comparably in both cells [5], and both basal and elevated levels of *CIT2* expression are dependent on Rtg1/Rtg3 [7,8]. Moreover, the transcripts encoding the TCA cycle and glycolytic enzymes were also increased in petite cells under repressing (*i.e.*, glucose) and derepressing (*i.e.*, raffinose) growth conditions, and the transcription requires Rtg1 and Rtg3 [9]. 

The RTG response is also modulated by several other pathways that regulate nutritional status and stress responses, such as the target of rapamycin (TOR) [10,11,12,13,14] and the Ras pathway [15]. Glutamate [16], glutamine [12] and proline [11] were reported as repressive signals for RTG activity, and both TOR-dependent and TOR-independent modes for RTG target gene expression have been demonstrated. The interplay between RTG response with other pathways, as well as the heterodimeric nature of Rtg1/Rtg3 regulators suggest that more complex metabolic regulations exist corresponding to various nutrition and growth conditions. In particular, metabolic signals that regulate RTG target genes are only partly understood, and it is unclear if the metabolites themselves are regulated by these pathways. Therefore, the characterization of metabolite pools would represent the first screening step to identify these metabolic signals.

In this study, we applied a metabolomics approach to find metabolic regulations possibly mediated by Rtg1 and Rtg3. Metabolomics, the exhaustive profiling of metabolites contained in a cell or an organism [17,18], has emerged as a powerful tool to study phenotypic changes. Metabolomics offers an unbiased view on cellular functions and links between genotype and phenotype [17]. Metabolic profiling can sensitively reveal silent genes [19,20] and is used for the prediction of complex and combinatorial phenotypes, such as lifespan [21]. Furthermore, the usefulness of the metabolome analysis of single-gene deletion mutants for understanding *S. cerevisiae*’s physiology has been demonstrated [22,23]. We found that while RTG gene deletion exhibited no difference in growth rates when grown in synthetic complete media, significant alteration in metabolic pathways, especially those involving polyamine biosynthesis, as well as TCA and glyoxylate cycles was observed. Our data showed that metabolic alterations occur at various metabolic sites and that these changes relate to different growth phases, but the difference can be detected even at the mid-exponential phase when mitochondrial function is repressed. Our study illustrates a broader assessment of metabolic change following RTG gene deletion than previously described.

## 2. Results and Discussion

### 2.1. Time-Course Metabolic Profiling of RTG-Deleted Strains

We performed time-course metabolic profiling of *RTG1* and *RTG3* deletion mutants to investigate the possible regulation of these transcription factors towards metabolic levels. The knock-out mutants were derived from the BY4742 wild-type strain available from European Saccharomyces Cerevisiae Archive for Functional Analysis (EUROSCARF) collection, by replacing the target gene with the *kanMX* cassette, which confers resistance against geneticin. Importantly, the gene deletion does not cause a growth defect in synthetic medium supplemented with essential amino acids, as there was no significant difference in maximum specific growth rates, µ of wild-type *vs.* mutants (µ = 0.456 ± 0.011 h^−^^1^, 0.461 ± 0.015 h^−1^ and 0.462 ± 0.013 h^−1^ for BY4742, *rtg1∆* and *rtg3∆*, respectively). Since RTG response depends on mitochondrial function and glyoxylate cycle regulation, we expected that the adaptation from glycolysis to gluconeogenesis in post-diauxic and stationary growth phases would yield sufficient metabolomics pattern, which can distinguish between WT and strains lacking the RTG response. Moreover, we expected that such a response against growth adjustment can be sensed at metabolite levels sooner, before there is a detectable change in phenotype, such as demonstrated previously in a yeast replicative lifespan study [21], prompting us to perform sampling at earlier growth phases, as well.

The yeast strains were cultured in a synthetic complete medium with 2% glucose as a carbon source, starting at OD_600_ = 0.1 (10^6^ cell/mL) for time 0 h. The cultures were grown to the stationary phase and sampled at four sampling points for metabolite measurement (Supplementary Figure S1). Each sampling point was taken at various times with different optical density values, OD_600_, corresponding to different growth phases; OD_600_ = 1 (10^7^ cells/mL) at 5 h for mid-exponential, OD_600_=5 (5 × 10^7^ cells/mL) at 9 h for late-exponential, OD_600_ = 10 (10^8^ cells/mL) at 26 h for post diauxic and at 76 h for stationary phases. The collected culture volume was adjusted according to the actual OD value, so that the total cell amount for metabolomics profiling is kept constant. Under high glucose condition, initially, *S. cerevisiae* operates mainly in the glycolytic mode to ferment glucose to ethanol independent of the presence of oxygen. During this stage, the expression of the genes encoding TCA cycle enzymes and other genes required for growth under non-fermentable carbon sources is repressed, a phenomenon known as glucose repression. Mitochondrial function is also repressed. Along with decreased glucose concentration, cells switch to gluconeogenesis and increase their respiratory rate and, finally, enter the stationary phase, where they accumulate storage carbohydrates. Therefore, our sampling points cover different metabolic states of the cells.

The identity of metabolites contained in yeast extracts was determined based on our in-house metabolite library. The identity was checked by spiking authentic standards to yeast extract and confirming that the particular metabolite peak intensity increases with added concentration. Pooled yeast aliquots were used as a quality control for reproducibility monitoring [24,25]. Peaks with poor reproducibility (relative standard deviation, RSD of peak intensity >30% [26]) were omitted from the list. Additionally, the peaks were normalized to an internal standard (see the Experimental Section), before being subjected to data analysis. As a result, we obtained 96 intracellular metabolites from yeast cell extracts (Supplementary Table S1).

**Figure 1:**
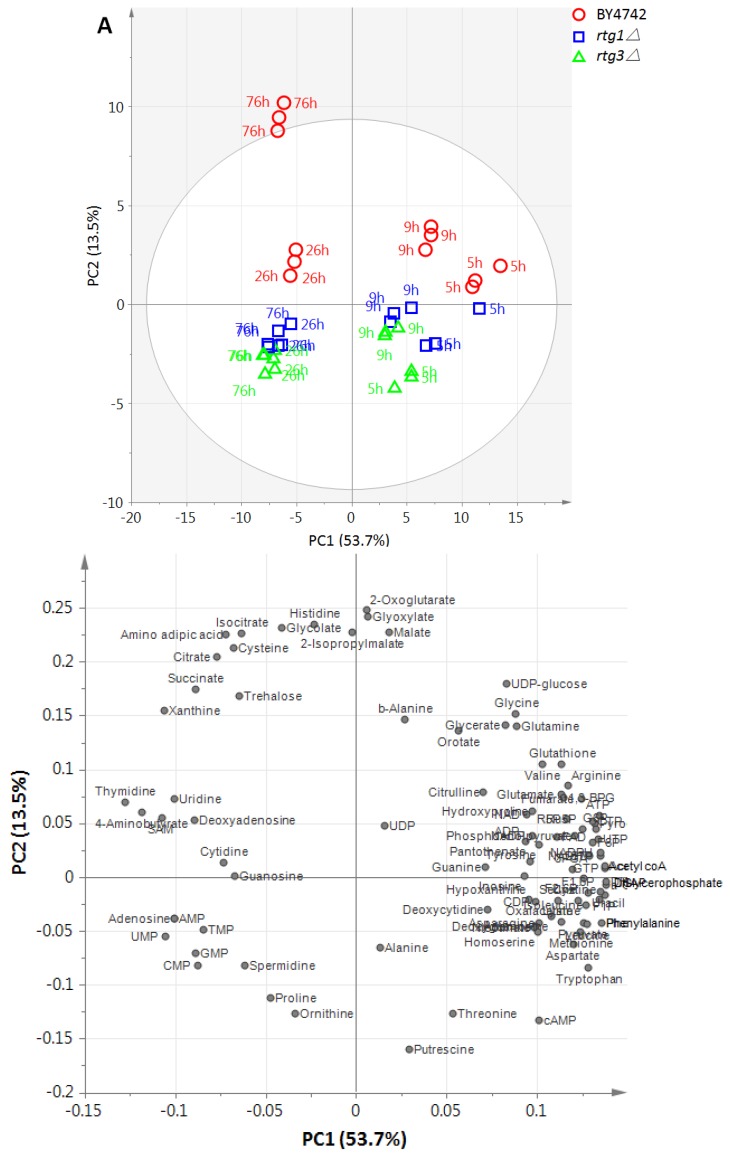
(**A**) PCA score plot for time-course metabolic profiling of wild-type strain BY4742 and the *rtg1∆* and *rtg3∆* mutants (*n =* 3). The metabolites were normalized to an internal standard and auto-scaled. The ellipse indicates the 95% confidence border based on Hotelling’s T^2^. Separation among different sampling points (different growth phases) can be seen along PC1, while the separation between WT and mutant strains was observable on PC2. (**B**) The corresponding loading plot illustrating metabolites that contribute to the separation on PC1 and PC2 (see Supplementary Table S2 for the loading values).

We employed principal component analysis (PCA), which is an unsupervised multivariate analysis method to reveal metabolic patterns between mutant and parental strains, as our first screening tool. Metabolome data (peak intensities normalized to an internal standard, mean-centered and scaled to unit variance) were fitted into PCA with five significant components. The PCA score plot (Figure 1A) shows that the first principal component (PC1, accounting for 53.7% of the total variance) separates between different growth phases, while Principal Component 2 (PC2, accounting for 13.5% of the total variance) separates between WT and mutant strains. This result indicated that gene deletion effects can be observed at metabolite levels with high resolution, even when there is no observable change in the growth rate.

We next examined the PCA loading plot (Figure 1B), which shows metabolites that contribute to the separation observed on the score plot. Along PC1, nucleotide monophosphates and ribonucleosides were seen as major contributors to the discrimination of samples at late growth phases (26 and 76 h), while proteinogenic amino acids, except for proline and cysteine, and glycolysis intermediates were generally abundant in samples at early growth phases. Along PC2, an increased level of 2-oxoglutarate and glyoxylate was distinctive in WT at 76 h, while putrescine, cAMP, threonine and ornithine were high in RTG-deficient strains. 

### 2.2. Metabolites and Metabolic Pathways Associated with RTG1 and RTG3

**Figure 2:**
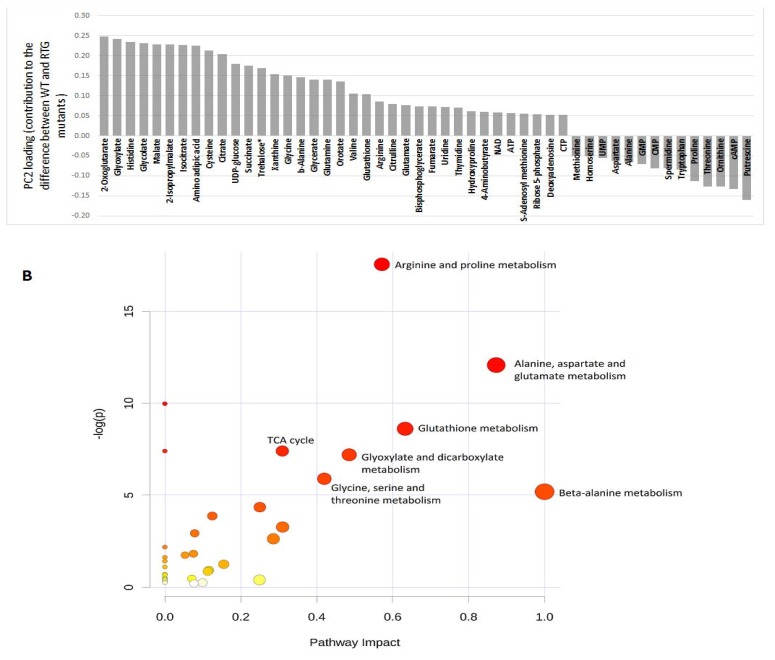
(**A**) Loading values of the 50 most influential metabolites along Principal Component 2, PC2, that distinguish mitochondrial retrograde (RTG) response-deficient strains from BY4742. These values indicate the contribution of each metabolite to the observed separation between WT and RTG mutants and the direction of each contribution. The upper and lower loading values were set at above 0.05 and below −0.05, respectively. Positive loadings indicate a positive correlation with RTG regulation (decreased when the RTG regulatory gene was deleted), while negative loadings indicate a negative correlation with RTG regulation (increased when the RTG regulatory gene was deleted). (**B**) Overview of pathway analysis, showing matched pathways according to pathway enrichment analysis and pathway impact values from pathway topology analysis. The circles represent the metabolite-matched pathways of *S. cerevisiae* retrieved from Kyoto Encyclopedia of Genes and Genomes (KEGG). The color intensity indicates the significance of the pathway, while size indicates the pathway impact score (the centrality of its involved metabolites). A complete list of pathway analysis results can be found in Supplementary Table S3.

As the difference between wild-type and RTG-deficient mutants can be seen as the second largest variation, we examined the metabolites that showed large loading values on PC2. The loadings describe the variable correlation to each PC. The 50 most important metabolites, with absolute loading values ≥0.05, are shown in Figure 2A. High levels of TCA and glyoxylate cycle intermediates (2-oxoglutarate, glyoxylate, malate, isocitrate, citrate, succinate) positively correlate with RTG genes (increased in BY4742 and decreased when RTG genes were deleted), while high levels of polyamine biosynthetic intermediates (putrescine, ornithine, spermidine) negatively correlate with RTG genes (increased when RTG genes were deleted). 

To get the overall view of the contribution of these metabolites into different metabolic pathways, we subjected the 50 most influential metabolites into the pathway analysis using MetaboAnalyst 2.0 [27]. Figure 2B summarizes the metabolic pathways affected by *RTG1* and *RTG3* deletion. Besides the TCA and glyoxylate cycles, amino acid metabolism makes up the majority of the affected pathways. Our result suggests that Rtg1 and Rtg3 may also hold regulatory effects on amino acid metabolism other than glutamate and glutamine.

**Figure 3:**
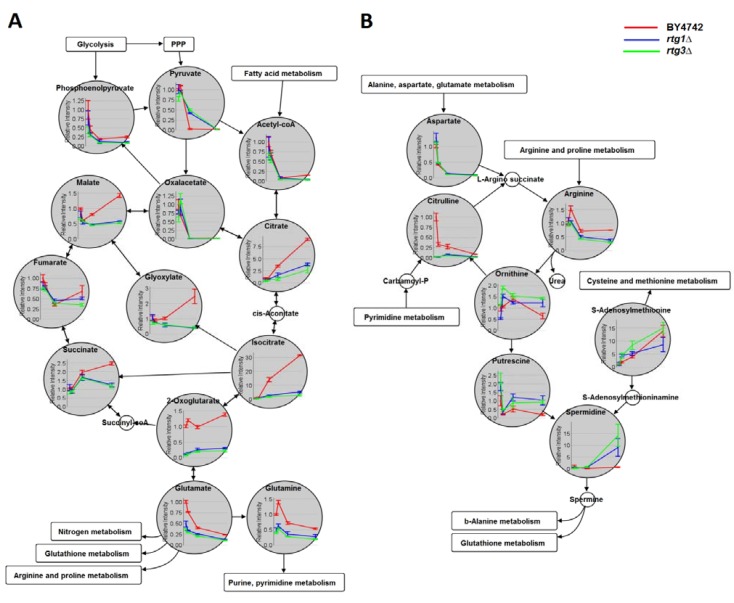
Time-course metabolic profiles of BY4742, *rtg1∆* and *rtg3∆* (*n =* 3) in (**A**) the TCA/glyoxylate cycle and (**B**) the superpathway of polyamine biosynthesis, shown together with the neighboring metabolic pathways (PPP: pentose phosphate pathway). These two pathways were the most affected by the deletion of the *RTG1* and *RTG3* genes. Metabolite intensities shown in the y-axis were normalized to an internal standard and relative to those of wild-type (BY4742) at time 5 h (OD_600_ = 1). In *S. cerevisiae*, the TCA cycle occurs in the mitochondria, while the glyoxylate cycle in the peroxisome; however, both are drawn as combined in this figure, since only bulk metabolites were measured. Note that in the event of fermentative metabolism and glyoxylate cycle activation, the flow from succinate to oxaloacetate is blocked (explaining the decreased levels of fumarate, which cannot be supplemented through the anaplerotic pathway).

Since the highest positive and negative loading was observed in 2-oxoglutarate and putrescine, respectively, we focused on the regulatory effects of Rtg1/Rtg3 on the TCA/glyoxylate cycle and the superpathway of polyamine biosynthesis. The time-course profiles of these metabolites are shown in Figure 3. As expected, citrate levels were reduced significantly in mutant strains, consistent with previous studies that reported the transcriptional regulation of *CIT2* by the Rtg1/Rtg3 complex [8]. Other metabolic intermediates shared in TCA and glyoxylate cycles (2-oxoglutarate, isocitrate, glyoxylate, malate, succinate) were also decreased in mutant strains, especially during deceleration/post-diauxic and stationary phases (Figure 3A). Fumarate, which is exclusive to the TCA cycle, showed no significant difference. These observations can be explained according to the different growth phases. Initially, yeast cells were under a fermentative (glucose repressing) condition, during which the TCA cycle and mitochondrial biogenesis are repressed [28]. As glucose concentration decreases, the cells prepare for the reversion of metabolic fluxes; reducing glycolytic activity and increasing the flux thorough the glyoxylate cycle and gluconeogenesis [29]. Glucose exhaustion leads to a transient diauxic phase, which induces gene transcription for mitochondrial proteins and adaptation to respiratory metabolism [30]. The low levels of TCA/glyoxylate cycle intermediates in RTG deletion mutants after the post-diauxic phase thus reflect the inability of the cells to supply anaplerotic citrate from the glyoxylate cycle, since the expression of *CIT2* requires Rtg1/3. Interestingly, 2-oxoglutarate showed a significant decrease from the mid-exponential phase (OD_600_ = 1).

Moreover, we observed elevated levels of polyamines putrescine and spermidine in *rtg1∆* and *rtg3∆* mutant strains at the stationary phase (the intensities of spermine from yeast extract were too low and could not be measured reliably, Figure 3B). Polyamine compounds have been associated with cytoprotective effects against oxidative and inflammatory stresses, and its depletion has been linked to yeast aging and necrosis [31,32]. However, other stress response-related metabolites, such as glutathione and trehalose, showed the opposite trend (lower in *rtg1∆* and *rtg3∆* at the stationary phase, Table 1). It is possible that polyamines might serve as defense metabolites against stresses when the RTG pathway is inactivated.

**Table 1:** Metabolite fold-change for the 50 most important metabolites for the *rtg1∆* and *rtg3∆* strains relative to wild-type strain BY4742 at each sampling time (inversed in the case of downregulation). Bold values indicate a statistically significant difference between *rtg1∆* and *rtg3∆* (*p* < 0.05, determined by a two-tailed heteroscedastic *t*-test and corrected for multiple testing (Benjamini and Hochberg false discovery rate, FDR).

Metabolites	5 h					9 h						26 h						76 h						
	*rtg1∆*				*rtg3∆*	*p*	*rtg1∆*			*rtg3∆*	*p*		*rtg1∆*		*rtg3∆*		*p*		*rtg1∆*		*rtg3∆*		*p*	
**TCA/glyoxylate cycle**																								
2-Oxoglutarate	−9.26				−15.92	0.179	−8.32			−9.48	0.682		−3.79		−5.07		0.205		−4.54		−6.28		0.356	
Malate	−1.23				−1.51	0.308	−1.14			−1.01	0.647		−1.69		−1.76		0.434		−2.45		−2.68		0.273	
Isocitrate	−3.47				−3.89	0.679	−1.17			−1.55	0.721		−5.15		−7.50		0.200		−5.94		−10.86		0.264	
Citrate	−1.69				−2.73	0.363	−1.37			−1.51	0.686		−2.11		−4.42		0.206		−2.35		−3.31		0.306	
Succinate	1.09				−1.25	0.358	−1.20			−1.21	0.980		−1.16		−1.19		0.830		−1.97		−2.11		0.530	
Fumarate	−1.23				−1.35	0.377	−1.10			−1.16	0.699		1.29		1.12		0.351		−1.34		−1.94		0.304	
Glyoxylate	1.09				−1.39	0.352	−1.21			−1.26	0.620		−1.77		−1.75		0.981		−6.40		−6.00		0.792	
Glycolate	−1.20			−1.22		0.885		−1.31	−1.23			0.665		−1.23		−1.06		0.236		−5.50		−6.52	0.376	
**Starch and sucrose metabolism**																								
UDP−glucose	−1.15			−1.46		0.377		−1.20	−1.34			0.719		−1.21		−1.56		0.332		−1.25		−1.50	0.330	
Trehalose	−1.08			−1.11		0.680		1.17	1.07			0.537		1.29		1.04		0.303		−2.23		−2.70	0.362	
**Pyrimidine metabolism**																								
Orotate	−1.40			−2.21		0.341		−1.25	−1.39			0.668		1.05		1.01		0.622		−1.08		−1.15	0.733	
Uridine	−1.48			1.18		0.385		1.31	1.96			0.659		−1.24		−1.26		0.987		−1.03		−1.05	0.907	
Thymidine	−1.19			1.03		0.376		−1.02	1.03			0.954		1.26		1.26		0.982		−1.26		−1.32	0.802	
CTP	−1.27			−1.55		0.363		−1.34	−1.36			0.953		−1.84		−3.12		0.214		−3.51		−4.69	0.341	
UMP	−1.28			−1.72		0.398		−1.44	−1.37			0.819		1.26		1.60		0.333		2.12		1.78	0.336	
CMP	−1.17			−1.23		0.867		−1.25	−1.25			0.999		1.71		1.77		0.831		2.25		2.16	0.808	
**Purine metabolism**																								
Xanthine	−1.08			1.58		0.374		−1.08	1.14			0.646		−1.24		−1.51		0.217		−1.92		−2.65	0.376	
ATP	−1.14			−1.33		0.385		−1.24	−1.37			0.660		−1.38		−2.64		0.196		−2.31		−3.81	0.298	
Deoxyadenosine	−1.57			−1.77		0.589		−1.04	1.10			0.675		1.24		1.36		0.325		−1.28		−1.17	0.434	
GMP	−1.15			−1.82		0.390		−1.06	−1.05			0.938		1.41		1.95		0.232		2.64		2.34	0.522	
cAMP	−1.02			1.03		0.864		1.20	1.29			0.787		1.01		1.63		0.287		4.27		4.43	0.800	
**Amino acid metabolism**																								
*Histidine metabolism*																								
Histidine	−1.18			−1.34		0.339		−1.30	−1.50			0.586		−2.52		−2.46		0.851		−2.62		−2.24	0.264	
*Cysteine and methionine metabolism*																								
Cysteine	−1.50			−2.62		0.210		−1.11	1.07			0.570		1.17		1.03		0.505		−3.34		−3.58	0.800	
Methionine	−1.11			−1.34		0.312		1.26	1.14			0.651		1.03		1.16		0.228		3.87		4.03	0.539	
S−Adenosylmethionine	1.15			1.02		0.840		2.40	2.29			0.924		1.33		2.12		0.212		−1.62		1.08	0.352	
*Valine*, *leucine and isoleucine metabolism*																								
2−Isopropylmalate	−1.54			−2.13		0.377		−2.07	−2.48			0.735		−1.37		−1.66		0.380		−1.64		−1.87	0.434	
Valine	−1.28			−1.58		0.235		−1.24	−1.36			0.795		−1.09		1.00		0.281		−1.26		−1.24	0.797	
*Lysine metabolism*																								
Amino adipic acid	−5.14			−11.67		0.369		−12.36	−14.42			0.644		−2.47		−3.68		0.190		−5.15		−7.05	0.339	
*Glycine*, *serine and threonine metabolism*																								
Glycine	−1.24			−1.64		0.354		−1.26	−1.18			0.812		−1.31		−1.25		0.835		−1.32		−1.36	0.588	
Glycerate	−1.09			−1.23		0.547		−1.15	−1.19			0.936		−1.13		−1.01		0.340		−1.50		−1.85	0.288	
Homoserine	1.31			−1.05		0.342		1.08	1.36			0.687		1.09		−1.10		0.323		1.17		−1.16	0.320	
Threonine	1.18			−1.02		0.345		1.49	1.60			0.740		1.85		1.77		0.248		1.79		1.56	0.285	
*Beta-alanine metabolism*																								
b-Alanine	1.34			−1.17		0.384		−1.30	−2.01			0.673		1.07		1.26		0.836		−2.98		−2.72	0.798	
*Arginine and proline metabolism*																								
Arginine	1.09			−1.04		0.395		−1.41	−1.51			0.868		−1.43		−1.69		0.307		−2.00		−2.53	0.330	
Citrulline	−35.03			−37.33		0.842		−12.89	−10.94			0.669		−3.39		−5.10		0.209		−3.48		−4.42	0.595	
Hydroxyproline	−1.06			−1.61		0.090		−1.05	1.02			0.938		−1.08		−1.46		0.325		−1.69		−1.15	0.264	
4-Aminobutyrate	−4.65			−5.55		0.689		−1.76	−1.37			0.678		−2.28		−1.48		0.232		1.14		−1.05	0.636	
Proline		−1.34	−1.45			0.817		−1.42	−1.25			0.682		−1.17		1.08		0.215		2.91		3.78	0.302	
*Alanine*, *aspartate and glutamate metabolism*																								
Glutamine		−2.01	−2.53			0.306		−2.25	−2.62			0.718		−2.02		−2.61		0.328		−1.93		−2.84	0.340	
Glutamate		−2.04	−2.76			0.369		−2.27	−2.63			0.189		−1.57		−1.93		0.237		−1.94		−2.19	0.266	
Aspartate		1.27	1.13			0.387		1.13	1.16			0.678		1.01		−1.20		0.179		1.41		1.16	0.286	
Alanine		−1.05	−1.23			0.355		1.08	1.07			0.989		1.21		1.22		0.967		1.86		1.71	0.286	
*Phenylalanine*, *tyrosine and tryptophan metabolism*																								
Tryptophan		−1.11	−1.26			0.336		−1.08	−1.14			0.714		1.38		1.56		0.218		1.79		1.97	0.275	
*Glutathione metabolism*, *polyamine biosynthesis*																								
Glutathione		−1.21	−1.43			0.344		−1.21	−1.27			0.640		−1.03		−1.07		0.272		−1.36		−1.51	0.332	
Spermidine		−1.33	−1.74			0.674		−1.12	−1.37			0.675		3.01		3.56		0.641		13.24		20.61	0.383	
Ornithine		−**1.87**	**1.13**			**0.002**		1.45	1.81			0.110		−1.15		1.09		0.251		1.90		2.21	0.334	
Putrescine		1.83	2.32			0.343		1.75	2.13			0.723		2.28		1.68		0.324		5.33		4.85	0.732	
**Pentose phosphate pathway**																								
Ribose 5-phosphate		−1.41	−2.46			0.341		−1.41	−1.58			0.616		−1.98		−2.06		0.934		−2.67		−2.47	0.651	
**Glycolysis**																								
Bisphosphoglycerate		−1.23	−2.25			0.304		−1.64	−1.62			0.985		−1.92		−2.48		0.522		−3.12		−3.99	0.446	
**Others (co-factors)**																								
NAD		−1.20	−1.37			0.328		−1.23	−1.24			0.932		−1.39		−1.51		0.294		−1.29		−1.39	0.343	

### 2.3. Metabolic Alteration Levels in RTG1 and RTG3

**Figure 4:**
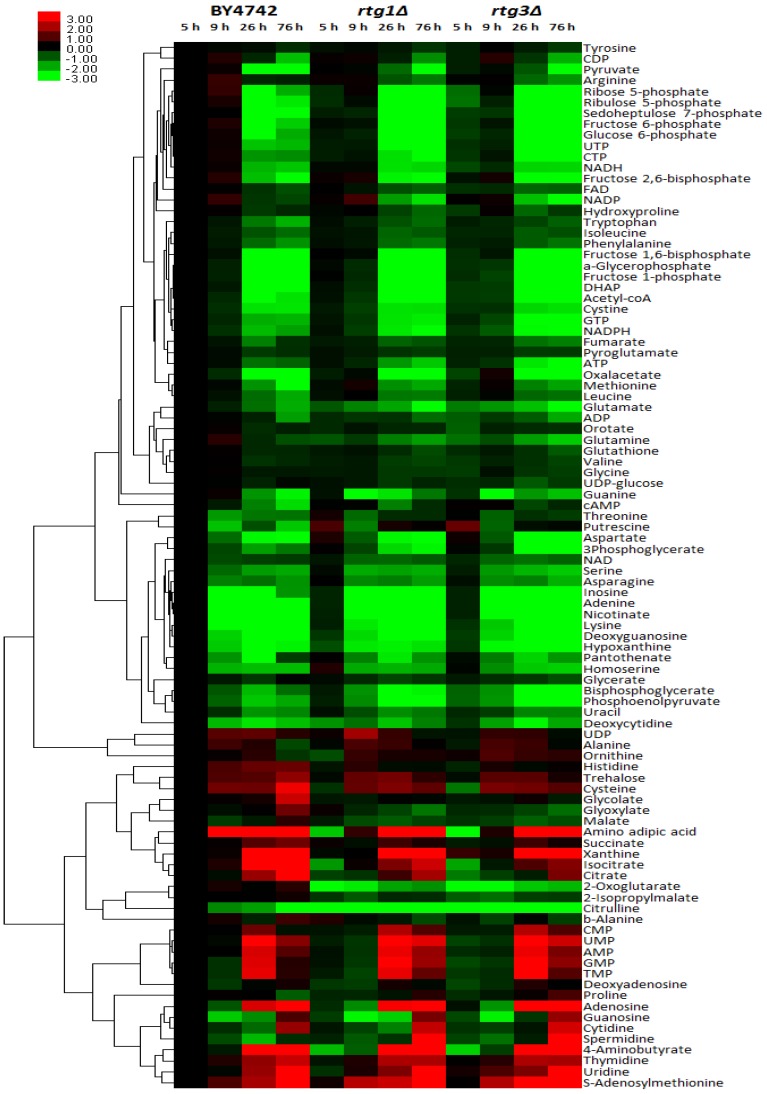
Heat map showing the differential metabolic expression in the BY4742, *rtg1∆* and *rtg3∆* strains at four different time points. Metabolite intensities were normalized to an internal standard and relative to those of wild-type BY4742 at time 5 h (OD_600_ = 1), averaged and log-2 transformed. Metabolite clustering was based on Pearson’s correlation and the average linkage (the distance between two clusters, x and y, is calculated as the average of all pairwise distances between the items contained in x and y).

We further sought whether *RTG1* and *RTG3* each exert a different degree of metabolic alteration. Since Rtg1 and Rtg3 act in a heterodimer complex and neither protein alone is able to bind R-box sites, it is anticipated that the deletion of either gene would result in a similar metabolic alteration. Figure 4 depicts a heat map of the metabolite changes of the BY4742, *rtg1∆* and *rtg3∆* strains at four different culture times, corresponding to different growth phases. Both *rtg1∆* and *rtg3∆* displayed a strikingly similar metabolic alteration pattern. In addition, fold-change values were calculated and the statistical difference between the two deletion strains was compared (Table 1). The fold-change values ranged from approximately 37-times (citrulline in *rtg3∆* at 5 h) to 21-times (spermidine in *rtg3∆* at 76 h). Only ornithine showed a significant difference between *rtg1∆* and *rtg3∆* at 5 h, while for the rest of the metabolites, *rtg1∆* and *rtg3∆* did not differ statistically across all time points. Therefore, we concluded that the deletion of either *RTG1* or *RTG3* yields in the same metabolic rearrangements, and the absence of either one component is sufficient for the loss of the RTG response. However, for a majority of metabolites, *RTG3* appears to have more profound effects on metabolic parameters (a larger fold-change) upon deletion than *RTG1*. This result was reflected in the PCA score plot (Figure 1A) where *rtg1∆* was positioned closer to BY4742 than *rtg3∆*.

### 2.4. Comparison with Previous Studies

The enzymatic and metabolic activities in *rtg1∆* and *rtg2∆* mutants have been described previously by Small *et al.* [33]. The authors reported that when compared to levels in the parental strain, the only changes seen, aside from the absence of peroxisomal citrate synthase, were a reduction in mitochondrial citrate synthase activity (~30%–50%), a reduction in acetyl-coA synthetase activity (~50%), a reduction in cytosolic (NAD) isocitrate dehydrogenase activity (~50%) and a reduction in pyruvate carboxylase activity (~50%). These enzymes are encoded by the genes, *CIT2*, *CIT1*, *ACS1/2*, *IDP2* and *PYC1/2*, respectively. While the reduced citrate and 2-oxoglutarate levels observed in this study might be explained by the reduced activity of citrate synthases and isocitrate dehydrogenase, we did not observe a difference in acetyl-coA and oxaloacetate levels between WT and RTG mutants. However, we noted that the experiment by Small *et al.* was conducted under a non-repressive condition, *i.e.*, using raffinose as a carbon source. Moreover, they indicated that the mutant cells have normally respiring mitochondria. In this study, we also confirmed that mitochondrial function is intact in *rtg1∆* and *rtg3∆*, as there were no difference in the cell counts of these strains, when grown on YPD (1% yeast extract, 2% peptone, 2% dextrose, 2% agar (% *w/v*)) *vs.* YPG (1% yeast extract, 2% peptone, 2% glycerol, 2% agar (% *w/v*)) plates (Supplementary Table S4).

In a recent large-scale microarray transcript profiling by Kemmeren *et al.* [34], they indicated that there is a marked decrease in *CIT1*, *CIT2*, *ACO1*, *IDH1* and *IDH2* expressions in *rtg1∆* and *rtg3∆* mutants. Similar to us, they used BY4742 derivatives and grew the yeast strains in synthetic complete (SC) medium with 2% glucose and sampled the cultures at the mid-exponential phase. Therefore, the reduced levels of citrate, isocitrate and 2-oxoglutarate observed in this study might be attributed to the reduced expression of *CIT1/2*, *ACO1* and *IDH1/2*, respectively. Moreover, the use of a rich medium does not seem to overcome the lack of 2-oxogluratarate production in RTG mutants. Kemmeren *et al.* also showed that *GAP1* and *AGP1* were upregulated in *rtg1∆* and *rtg3∆*. Gap1 is a general amino acid permease that directs the uptake of all naturally occurring amino acids [35,36] and has been reported to be regulated by the nitrogen source and amino acid levels [37], while Agp1 is an amino acid permease, which transports asparagine and glutamine [38]. In this study, while the endogenous glutamate level was significantly lower in RTG mutants, the exogenous glutamate concentration in the medium for all strains did not differ (Supplementary Figure S2). However, reduced levels of both intracellular and extracellular glutamine in RTG mutants can be seen (Supplementary Figure S2). Why increased Gap1 and Agp1 induced by *RTG1/3* deletion seemed to result in an increased uptake of extracellular glutamine, but not glutamate, is not clear. Interestingly, two genes that encode glutamate dehydrogenases for the synthesis (*GDH1*) and degradation (*GDH2*) of glutamate were also increased in RTG mutants. Taken together, several explanations may underlie these observations; (1) there’s a limit on the uptake level of glutamate when it is abundantly present in the growth medium; (2) glutamine is preferred over glutamate for an uptake into the cells; and (3) 2-oxoglutarate accumulation is primarily governed by *de novo* synthesis from isocitrate by *IDH1/2* and not much from glutamate degradation by *GDH2*. 

The characteristic decrease in 2-oxogutarate concentrations in *rtg1∆* and *rtg3∆* during the mid-exponential growth phase suggests that this metabolite might play a critical role in controlling the flow and balance of TCA/glyoxylate cycles. Further experiments, e.g., a flux analysis using labeled substrates, should be performed to confirm the origin of 2-oxoglutarate under sufficient glutamate/glutamine concentrations in the growth medium and to investigate the physiological attributions of this metabolite to the metabolic reprogramming under RTG deletion.

### 2.5. Yeast Chronological Lifespan and Its Relation with RTG1 and RTG3

**Figure 5:**
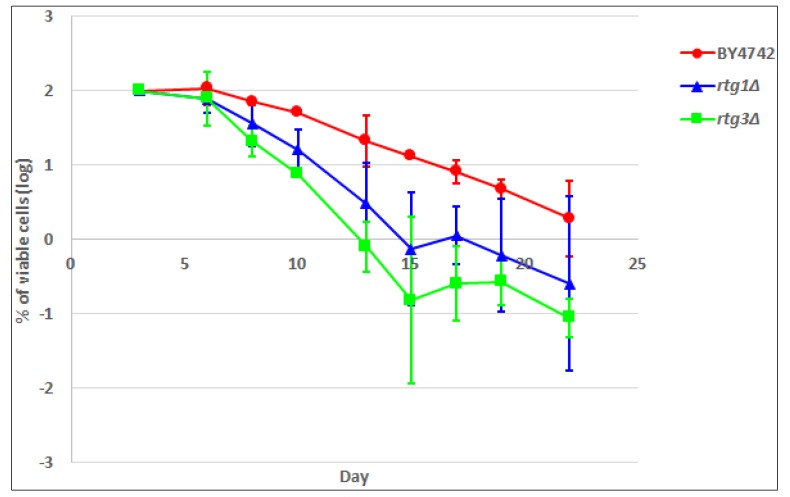
The chronological lifespan (CLS) of BY4742, *rtg1∆* and *rtg3∆*, measured as the number (log %) of viable cells in exhausted growth media after revival on YPD (1% yeast extract, 2% peptone, 2% dextrose, 2% agar (% *w/v*)) plates. CLS at Day 3 (start of the stationary phase) was defined as 100%.

The effect of metabolic rearrangements following RTG response activation/deactivation can be evaluated in terms of yeast lifespan. Activation of the mitochondrial RTG pathway has been reported to contribute to genome stability [39] and to increase the yeast chronological lifespan, CLS [40]. In a separate study, decreased TOR signaling was also shown to extend CLS [41]. CLS is the period of time in which cells remain viable in a non-dividing state after nutrients cease in the stationary phase [42], often expressed as the number of colonies recovered when the yeast cells are transferred back to a growth allowing environment. In this study, we measured the CLS of wild-type BY4742, *rtg1∆* and *rtg3∆* (Figure 5). Although there were some overlaps among the three strains at Day 19 and Day 22, *rtg1∆* and *rtg3∆* showed the tendency of a shorter CLS compared with WT. Together with metabolome data (Table 1 and Supplementary Table S1), several observations can be made. Trehalose and glutathione, two metabolites that have been positively related to stress response, were accumulated in BY4742 at the stationary phase and, thus, might have contributed to the extended CLS in the wild-type strain. Intracellular amino acids, such as histidine, glycine, glutamine, valine, arginine and glutamate, were lower, while methionine, aspartate, alanine, tryptophan, proline and threonine were higher at the stationary phase in deletion strains. The addition of isoleucine, threonine and valine to growth media was reported to extend CLS [43]; however, the effects of the intracellular amino acid level were not clarified. Analysis of growth media (Supplementary Figure S3) revealed that extracellular threonine and valine were high in BY4742. 

## 3. Experimental Section

### 3.1. Strain Growth Conditions and Sample Preparation

Yeast BY4742 (*MATα leu2∆0 lys2∆0 ura3∆0 his3∆1*) and its isogenic derivatives, *rtg1Δ* (*MATα leu2∆0 lys2∆0 ura3∆0 his3∆1 rtg1*::*kanMX*) and *rtg3Δ* (*MATα leu2∆0 lys2∆0 ura3∆0 his3∆1 rtg3*::*kanMX*), were purchased from Open Biosystems (Huntsville, AL, USA). All cultures were grown in synthetic media composed of 0.67% Difco™ yeast nitrogen base w/o amino acids (BD, MD, USA), 2% glucose and standard concentrations of amino acids and bases [44]. The cultivation and sampling were performed as described previously [23]. Briefly, pre-cultured yeast cells were grown at 30 °C in a rotary shaker (200 rpm) to desired optical densities at 600 nm (OD_600_) and harvested using a rapid filtration system. The harvested cell amount was kept at 5 OD units, equivalent to 1 mg of cells by dry weight at each sampling point. After washing with 5 mL water, the filter-bound cells were inserted into 1 mL of −30 °C precooled single-phase extraction solvent (methanol/chloroform/water = 5/2/2 *v/v/v* %) with added 1.2 µg/mL of 1,4-piperazinediethanesulfonic acid (Dojindo, Kumamoto, Japan) as an internal standard and immediately quenched in liquid nitrogen. The samples were kept at −80 °C until extraction. Extraction was carried out at 4 °C, 1,200 rpm for 30 min. After that, all liquid extract was transferred to a new tube, 400 µL water added, vortexed and centrifuged at 4 °C, 16,100 rcf (relative centrifugal force) for 3 min to separate polar and non-polar phases. The upper polar phase was collected, concentrated five times from the initial volume and ready for LC-MS analysis. Extracted samples were analyzed within 24 h after extraction. 

For extracellular metabolome, ~1 mL of the medium filtrate was collected at the same time during cell filtration and diluted four times with water prior to LC-MS analysis.

### 3.2. Metabolite Profiling and Quantification

The analysis platform consists of a Shimadzu Nexera series UHPLC system (Shimadzu, Kyoto, Japan) coupled to a triple quadrupole mass spectrometer, LCMS-8030, with a modification to improve the sensitivity (Shimadzu, Kyoto, Japan). Two LC/MS methods were employed; (1) ion-pairing reversed phase ultrahigh performance liquid chromatography-tandem mass spectrometry (UHPLC-MS/MS) to detect mainly anionic metabolites, such as sugar phosphates and nucleotides from primary metabolism; and (2) regular reversed phase UHPLC-MS/MS for the analysis of other metabolites undetected in ESI negative mode. Ion-pairing UHPLC-MS/MS was performed as described previously [23]. For regular reversed phase UHPLC-MS/MS, the parameters were as follows: column: Discovery HS F5-3 (150 mm × 2.1 mm, 3 µm, Supelco Analytical, PA, USA); flow rate: 0.3 mL/min; column temperature: 40 °C; mobile Phase A: water with 0.1% formic acid; mobile Phase B: acetonitrile with 0.1% formic acid; gradient program: 0% B (0–1 min)–20% B (11 min)–100% B (11.5–13 min)–0% B (13.1–15 min); sample cycle time: 15 min; injection volume: 3 µL. The mass spectrometric parameters were: ESI positive mode; desolvation line (DL) temperature: 250 °C; nebulizer gas flow: 2 L/min; heat block temperature: 400 °C; other parameters were optimized automatically by flow injection analysis and auto-tuning. The optimized multiple reaction monitoring (MRM) parameters and retention time for each metabolite are listed in Supplementary Table S5.

All samples were kept in a 4 °C autosampler during analysis. Standard mixtures of authentic metabolites and the pooled QC sample [25,26] were injected periodically throughout the analysis run for evaluating the stability and reproducibility of the analytical system. All reagents were of LC-MS grades (Wako, Osaka, Japan).

Peak picking was conducted by LabSolutions (Shimadzu, Kyoto, Japan) followed by manual inspection. The parameters were set as follows: integration: auto, max peak: 3, width: 5 s; smoothing: standard, counts: 5, width: 1 s; identification: absolute RT and closest peak, target window: 5%, reference window: 5%, process time: ±1 min. Obtained peaks were identified based on an in-house metabolite library and confirmed by the spiking of authentic standards.

### 3.3. Multivariate Data Analysis

The amount of each metabolite (peak intensity) was normalized to internal standard, mean-centered and scaled to unit variance. Principal component analysis (PCA) was performed using SIMCA-P+ ver13 (Umetrics, Umeå, Sweden). Pathway analysis was performed using MetaboAnalyst 2.0 [27]. Heat map and hierarchical clustering of fold-change normalized intensities were performed on Cluster 3.0 [45] and viewed on Java Treeview [46]. The statistical difference (two-tailed heteroscedastic *t*-test) was calculated using MS Excel. Pathway mapping was performed by VANTED V2.1.0 [47].

### 3.4. Yeast Chronological Lifespan Measurement

The chronological lifespan (CLS) measurement was based on [48]. Briefly, aliquots of yeast culture grown to the stationary phase were diluted to approximately 10^3^–10^4^ cells/mL, and 100 µL were spread onto YPD (1% yeast extract, 2% peptone, 2% dextrose, 2% agar (% *w/v*)) plates. Yeast colonies were counted after 2–4 days of incubation at 30 °C. CLS (at Day X) is defined as the percentage of the number of colonies at Day X divided by the number of colonies at Day 3. 

## 4. Conclusions

In this study, metabolic profiling of *rtg1∆* and *rtg3∆* strains was performed. By relative comparison of the metabolic alteration in these deletion strains with wild-type strain BY4742 and multivariate data analysis, we could identify metabolites and metabolic pathways associated with *RTG1*/*RTG3* genes and possibly related to the mitochondrial RTG response. Besides the TCA and glyoxylate cycles, which have been identified previously, we found that various pathways, including amino acid metabolism, were affected. Of note were the markedly reduced 2-oxoglutarate level, which was detected at as early as the exponential phase, and elevated levels of polyamines at the stationary phase in *rtg1∆* and *rtg3∆*. We also discovered that *RTG3* deletion imposed greater effects on most metabolites other than *RTG1*. In addition, we confirmed that RTG gene deletion shortens the chronological lifespan (CLS) in yeast. Our data illustrate the power of metabolomics in finding gene/transcription factor-metabolite correlations and provides a broader assessment of metabolic change following RTG gene deletion. We expect that our study will lead to deeper investigations into RTG responses and bHLH proteins in general. 

## Supplementary Files

Supplementary File 1
