# Supplementary material for: Metabolic Profiling of Retrograde Pathway Transcription Factors Rtg1 and Rtg3 Knockout Yeast
Source: Metabolites. 2014 Jul 8;4(3):580–98. doi: 10.3390/metabo4030580 (PMC4192681; doi:10.3390/metabo4030580)
Supplement: Supplementary File 1 [file metabolites-04-00580-s001.zip › metabolites-04-00580-supplementary/metabolites-04-00580-supplementary.pdf]

Supplementary Information

Figure S1. Growth curve for BY4742, *rtg1*Δ and *rtg3*Δ under synthetic complete media (*n* = 3). Metabolome sampling was done at 5, 9, 26 and 76 h.

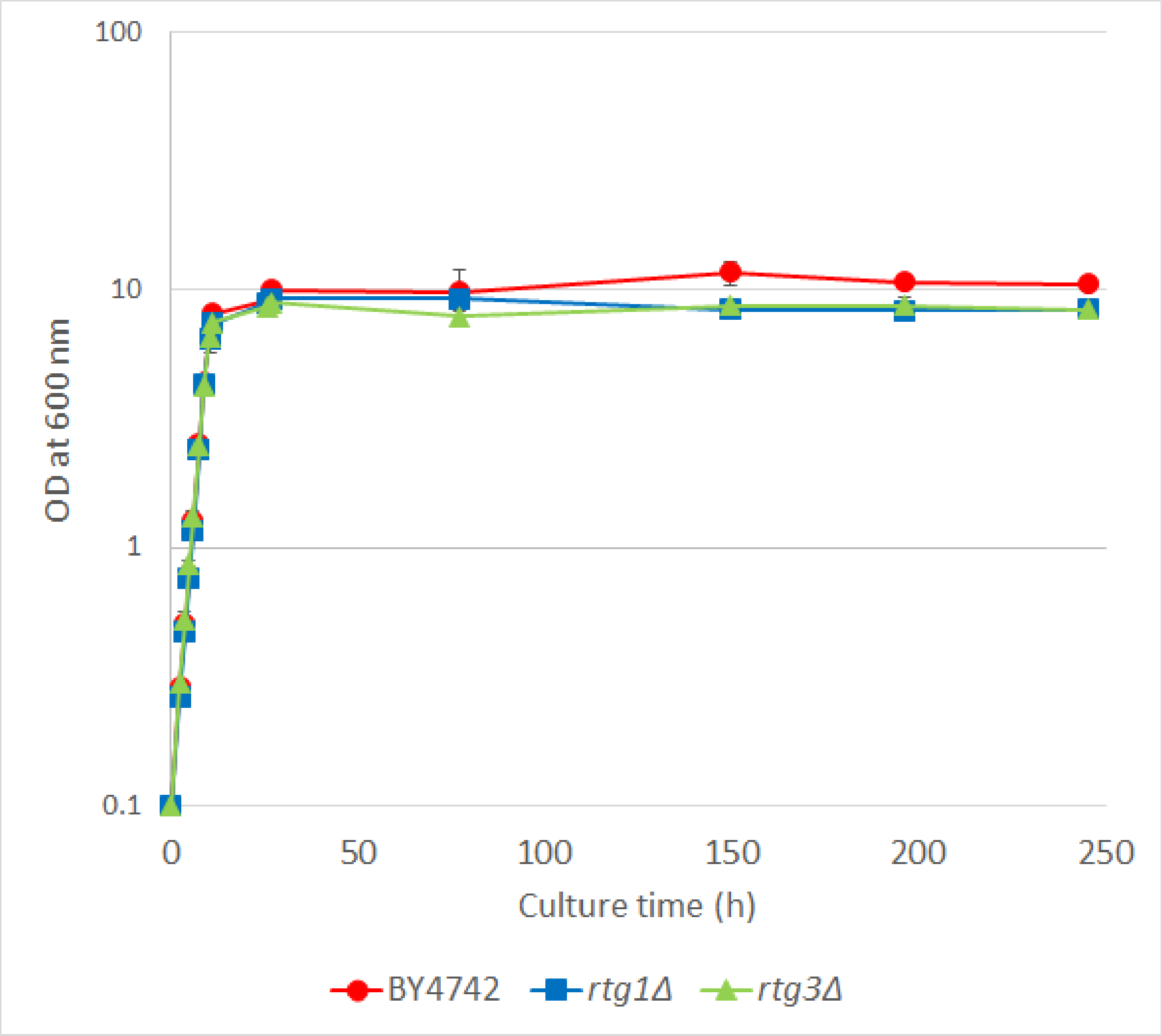

Supplementary Table S1 (Excel file). Raw data of metabolite intensities of BY4742, *rtg1*Δ and *rtg3*Δ strains. PIPES (1,4-piperazinediethanesulfonic acid) was used as an internal standard.

Table S2. Loading values on principal component 1 (PC1) and principal component 2 (PC2) for each metabolite (intracellular: from the yeast extract and extracellular: from the growth medium).

Metabolite (intracellular)	PC1 Loading	PC2 Loading	Metabolite (extracellular)	PC1 Loading	PC2 Loading
2-Isopropylmalate	–0.00209	0.22745	2-Isopropylmalate	–0.16400	0.07362
2-Oxoglutarate	0.00588	0.24828	2-Oxoglutarate	–0.11752	0.05733
3Phosphoglycerate	0.11976	0.00749	4-Aminobutyrate	–0.14398	0.14484
4-Aminobutyrate	–0.11829	0.05978	Adenine	0.14294	0.18411
Acetyl-coA	0.13741	0.01074	Adenosine	–0.12229	0.08622
Adenine	0.09936	–0.04542	a-Glycerophosphate	–0.17368	–0.02441
Adenosine	–0.10064	–0.03844	Alanine	0.11893	–0.04041
ADP	0.09370	0.03354	Amino adipic acid	–0.15262	0.14734
a-Glycerophosphate	0.13798	–0.00768	AMP	–0.15113	0.12424
Alanine	0.01347	–0.06582	Arginine	0.14229	0.18760
Amino adipic acid	–0.07215	0.22560	Asparagine	0.15189	0.16691
AMP	–0.10022	–0.03836	Aspartate	0.14460	0.18242
Arginine	0.11712	0.08488	Citrate	–0.13431	0.17177
Asparagine	0.10135	–0.04259	Cystine	0.10505	–0.10305
Aspartate	0.12016	–0.06230	Deoxyadenosine	–0.17569	0.00762
ATP	0.13473	0.05739	Deoxyguanosine	–0.12206	–0.18288
b-Alanine	0.02704	0.14669	DHAP	–0.14678	–0.07630
Bisphosphoglycerate	0.11485	0.07410	Glutamate	0.13630	0.20084
cAMP	0.10106	–0.13327	Glutamine	0.10919	0.22477
CDP	0.09910	–0.02309	Glutathione	–0.15461	0.13571
Citrate	–0.07668	0.20449	Glycine	0.04117	0.05761
Citrulline	0.07007	0.07924	Glycolate	–0.12834	0.16408
CMP	–0.08741	–0.08178	Glyoxylate	–0.08650	0.18133
CTP	0.13086	0.05169	Guanosine	–0.11670	0.07474
Cysteine	–0.06774	0.21267	Histidine	0.16084	0.04404
Cystine	0.13480	–0.02100	Hypoxanthine	0.13818	0.18256
Cytidine	–0.07314	0.01314	Inosine	–0.16559	–0.02855
Deoxyadenosine	–0.08935	0.05245	Isocitrate	–0.12434	0.17564
Deoxycytidine	0.07273	–0.02977	Leucine	0.17279	–0.06298
Deoxyguanosine	0.10825	–0.03625	Lysine	0.14266	0.18386
DHAP	0.13796	–0.00388	Malate	–0.17103	0.08345
FAD	0.11104	0.03701	Methionine	0.17403	–0.04695
Fructose 1,6-bisphosphate	0.13499	–0.01370	NAD	–0.14924	0.12477
Fructose 1-phosphate	0.13737	–0.01639	Nicotinate	0.14247	0.18098
Fructose 2,6-bisphosphate	0.12608	–0.00098	Orotate	–0.15549	–0.11610
Fructose 6-phosphate	0.13058	0.03167	Oxalacetate	0.06816	–0.15088
Fumarate	0.12440	0.07301	Pantothenate	–0.00519	0.26084
Glucose 6-phosphate	0.13294	0.04489	Phenylalanine	0.17340	0.02699
Glutamate	0.11327	0.07720	Phosphoenolpyruvate	–0.13665	0.16323

Table S2. *Cont.*

Metabolite (intracellular)	PC1 Loading	PC2 Loading	Metabolite (extracellular)	PC1 Loading	PC2 Loading
Glutamine	0.08882	0.13960	Proline	–0.13749	–0.02614
Glutathione	0.11373	0.10429	Pyroglutamate	0.03414	–0.02368
Glycerate	0.08263	0.14066	Pyruvate	0.05378	–0.24100
Glycine	0.08802	0.15103	Serine	0.15543	0.15707
Glycolate	–0.04132	0.23183	Succinate	–0.16794	0.08129
Glyoxylate	0.00639	0.24135	Threonine	0.11451	0.22466
GMP	–0.08884	–0.07027	Thymidine	–0.17320	0.04120
GTP	0.13770	0.00852	Trehalose	0.14937	–0.02877
Guanine	0.07134	0.00928	Tryptophan	0.15976	–0.13349
Guanosine	–0.06730	0.00043	Tyrosine	0.13203	0.15300
Histidine	–0.02338	0.23441	Uracil	0.07631	0.17593
Homoserine	0.10027	–0.05115	Uridine	–0.15427	0.09296
Hydroxyproline	0.09716	0.06096	Valine	0.02936	0.07420
Hypoxanthine	0.09569	–0.02078	Xanthine	–0.16286	0.10989
Inosine	0.09335	0.00044			
Isocitrate	–0.06356	0.22648			
Isoleucine	0.12724	–0.02595			
Leucine	0.12801	–0.04393			
Lysine	0.11374	–0.04160			
Malate	0.01816	0.22764			
Methionine	0.12383	–0.05087			
NAD	0.09416	0.05793			
NADH	0.12883	0.01969			
NADP	0.09754	0.03847			
NADPH	0.13540	0.02288			
Nicotinate	0.09883	–0.04632			
Ornithine	–0.03392	–0.12659			
Orotate	0.05678	0.13618			
Oxalacetate	0.12304	–0.02171			
Pantothenate	0.09640	0.01449			
Phenylalanine	0.13560	–0.04245			
Phosphoenolpyruvate	0.12118	0.03853			
Proline	–0.04735	–0.11264			
Putrescine	0.02963	–0.16010			
Pyroglutamate	0.13187	0.04943			
Pyruvate	0.12589	–0.04248			
Ribose 5-phosphate	0.11655	0.05339			
Ribulose 5-phosphate	0.12525	0.04431			
S-Adenosylmethionine	–0.10702	0.05528			
Sedoheptulose 7-phosphate	0.13532	0.02000			
Serine	0.11174	–0.02229			
Spermidine	–0.06151	–0.08182			
Succinate	–0.08868	0.17453			

Table S2. *Cont.*

Metabolite (intracellular)	PC1 Loading	PC2 Loading	Metabolite (extracellular)	PC1 Loading	PC2 Loading
Threonine	0.05368	–0.12654			
Thymidine	–0.12763	0.06990			
TMP	–0.08416	–0.04919			
Trehalose	–0.06462	0.16862			
Tryptophan	0.12819	–0.08465			
Tyrosine	0.10120	0.02960			
UDP	0.01583	0.04811			
UDP-glucose	0.08317	0.17915			
UMP	–0.10538	–0.05556			
Uracil	0.12805	–0.01441			
Uridine	–0.10055	0.07235			
UTP	0.13373	0.03512			
Valine	0.10324	0.10526			
Xanthine	–0.10614	0.15427			

Table S3. Pathway analysis using MetaboAnalyst 2.0 (hits ≥ 2, arranged according to *p*-values).

Pathway Name	Total	Hits	p	-log (p)	Holm p	FDR	Impact
Arginine and proline metabolism	37	13	2.37E-08	17.556	1.54E-06	1.54E-06	0.57168
Alanine, aspartate and glutamate metabolism	20	8	5.69E-06	12.077	0.000364	0.000185	0.87254
Aminoacyl-tRNA biosynthesis	67	13	4.65E-05	9.9767	0.002928	0.001007	0
Glutathione metabolism	23	7	0.000182	8.6137	0.011259	0.002951	0.63277
Citrate cycle (TCA cycle)	20	6	0.000608	7.4058	0.03707	0.006591	0.30939
Nitrogen metabolism	8	4	0.000608	7.4047	0.03707	0.006591	0
Glyoxylate and dicarboxylate metabolism	14	5	0.000747	7.1989	0.044096	0.00694	0.48551
Glycine, serine and threonine metabolism	26	6	0.00275	5.8963	0.15948	0.022342	0.41988
beta-Alanine metabolism	7	3	0.005565	5.1912	0.31722	0.040194	1
Pyrimidine metabolism	35	6	0.012898	4.3507	0.72228	0.083836	0.25014
Lysine biosynthesis	19	4	0.020822	3.8717	1	0.12304	0.125
Cysteine and methionine metabolism	33	5	0.037927	3.2721	1	0.20544	0.31009
Purine metabolism	60	7	0.053069	2.9362	1	0.26535	0.07859
Butanoate metabolism	17	3	0.071451	2.6387	1	0.33174	0.28571
Cyanoamino acid metabolism	10	2	0.11148	2.1939	1	0.48308	0
Valine, leucine and isoleucine biosynthesis	24	3	0.16013	1.8318	1	0.65052	0.07519
Sulfur metabolism	13	2	0.17308	1.754	1	0.66178	0.05319
Propanoate metabolism	14	2	0.19472	1.6362	1	0.70314	0
Pantothenate and CoA biosynthesis	16	2	0.23889	1.4318	1	0.81724	0
Starch and sucrose metabolism	18	2	0.28357	1.2603	1	0.92162	0.15497
Porphyrin and chlorophyll metabolism	20	2	0.32811	1.1144	1	1	0
Pyruvate metabolism	23	2	0.39349	0.93271	1	1	0.1159

Table S4. Cell growth on YPD (1% yeast extract, 2% peptone, 2% dextrose, 2% agar (% *w/v*)) and YPG (1% yeast extract, 2% peptone, 2% glycerol, 2% agar (% *w/v*)) plates, expressed as the number of colonies. Cell cultures were diluted to approximately 10^3 cells/ mL, 100 µL were spread on YPD or YPG plates, and the colony number was counted after 2-4 days. Measurement was done in duplicate (separated by a comma) for each sampling point.

Strain	Plate	Day 1	Day 3	Day 5
BY4742	YPD	86, 115	100, 100	68, 53
	YPG	81, 151	96, 112	66, 54
*rtg1*Δ	YPD	53, 52	31, 34	25, 28
	YPG	61, 62	39, 43	29, 28
*rtg3*Δ	YPD	23, 16	27, 45	6, 8
	YPG	28, 15	25, 40	10, 11

Figure S2. Intracellular (from cell extracts) and extracellular (from the growth medium) concentrations of glutamate and glutamine in BY4742, and *rtg1*Δ and *rtg3*Δ mutants (n = 3). Y-axis indicates relative intensity while x-axis indicates time. The metabolite intensities were relative to that of BY4742 at time 5 h.

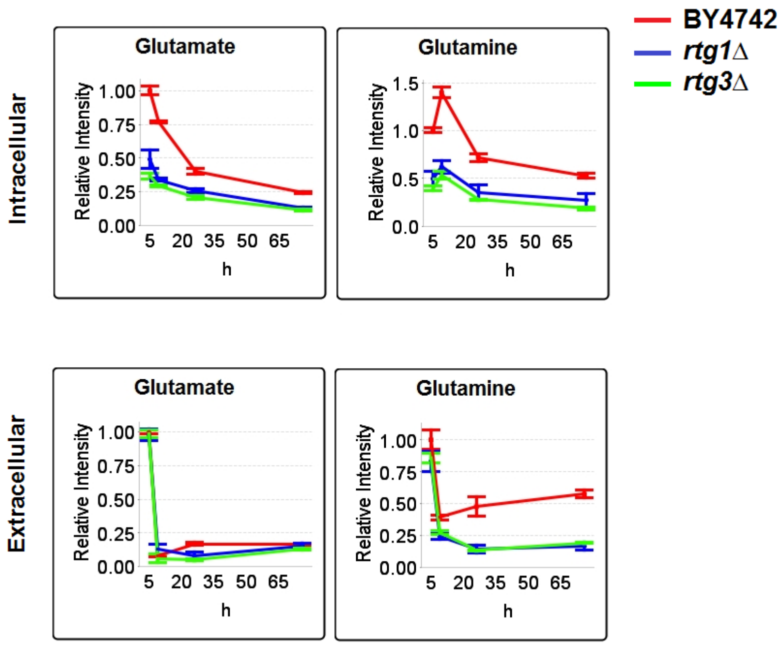

Figure S3. (**A**) PCA score plot for time-course extracellular metabolic profiling (from growth media) of wild type strain BY4742, and *rtg1*Δ and *rtg3*Δ mutants (n = 3). The metabolites were scaled to unit variance. Ellipse indicate 95% confidence border based on Hotelling's T^2; (**B**) The corresponding loading plot illustrating metabolites that contribute to the separation on PC1 and PC2 (see Supplementary Table S2 for the loading values).

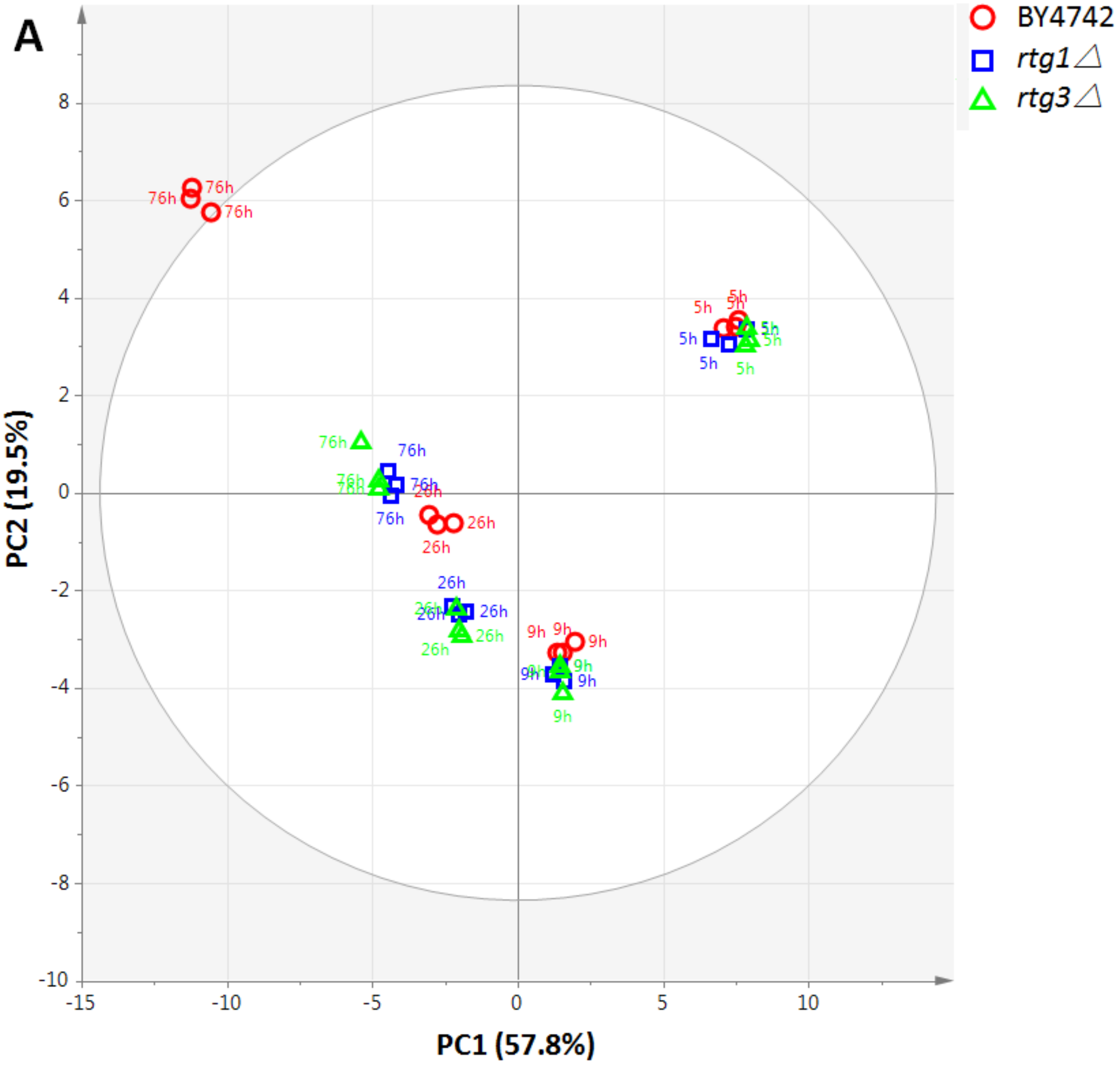

Figure S3. *Cont.*

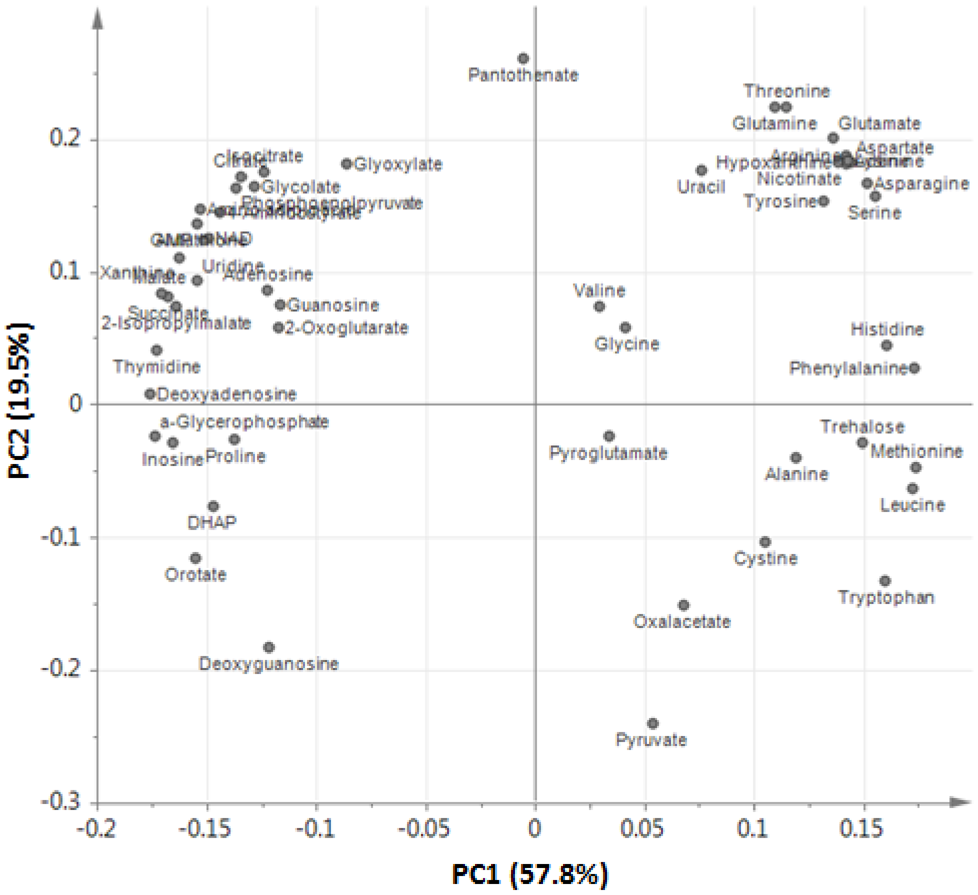

Table S5. Optimized multiple reaction monitoring (MRM) parameters and retention time for each metabolite.

No	Metabolite	LC-MS Method */mode	Precursor ion *m/z*	Product ion *m/z*	Retention Time (min)	Target Q1 Pre Bias (V)	Target Collision Energy (V)	Target Q3 Pre Bias (V)
1	Arginine	Ion-pair RP/ESI negative	173.10	131.20	1.039	13	15	24
2	Histidine	Ion-pair RP/ESI negative	154.00	93.15	1.038	12	21	16
3	Serine	Ion-pair RP/ESI negative	104.00	74.15	1.138	12	16	13
4	Asparagine	Ion-pair RP/ESI negative	131.00	113.15	1.151	10	15	21
5	Glutamine	Ion-pair RP/ESI negative	145.00	127.05	1.160	12	18	18

Table S5. *Cont.*

No	Metabolite	LC-MS Method */mode	Precursor ion *m/z*	Product ion *m/z*	Retention Time (min)	Target Q1 Pre Bias (V)	Target Collision Energy (V)	Target Q3 Pre Bias (V)
6	Threonine	Ion-pair RP/ESI negative	118.00	74.05	1.186	11	15	13
7	Trehalose	Ion-pair RP/ESI negative	341.00	89.00	1.302	15	23	16
8	Proline	Ion-pair RP/ESI negative	114.00	68.10	1.331	10	15	11
9	Methionine	Ion-pair RP/ESI negative	148.00	47.05	1.987	11	14	16
10	Isoleucine	Ion-pair RP/ESI negative	130.10	45.00	2.578	11	15	15
11	Adenine	Ion-pair RP/ESI negative	134.05	107.35	2.829	28	20	20
12	Tyrosine	Ion-pair RP/ESI negative	180.00	163.05	2.834	12	18	18
13	Xanthine	Ion-pair RP/ESI negative	151.20	108.15	3.000	16	20	19
14	Amino adipic acid	Ion-pair RP/ESI negative	160.00	116.20	3.171	12	17	21
15	Glutamate	Ion-pair RP/ESI negative	146.00	102.20	3.273	11	15	18
16	Aspartate	Ion-pair RP/ESI negative	132.00	88.05	3.467	10	14	15
17	Inosine	Ion-pair RP/ESI negative	267.00	135.15	4.559	21	23	24
18	Guanosine	Ion-pair RP/ESI negative	282.10	150.20	4.706	22	21	28
19	Phenylalanine	Ion-pair RP/ESI negative	164.00	103.15	4.854	13	18	19
20	Glycolate	Ion-pair RP/ESI negative	75.00	75.00	5.001	16	15	15
21	Glycerate	Ion-pair RP/ESI negative	105.00	75.15	5.088	12	15	26
22	Adenosine	Ion-pair RP/ESI negative	266.10	134.15	5.385	18	20	26
23	Glyoxylate	Ion-pair RP/ESI negative	73.00	73.00	5.577	14	13	15
24	Pyroglutamate	Ion-pair RP/ESI negative	128.00	84.10	6.438	10	14	15
25	Glucose 6-phosphate	Ion-pair RP/ESI negative	258.90	97.05	6.734	20	21	17

Table S5. *Cont.*

No	Metabolite	LC-MS Method */mode	Precursor ion *m/z*	Product ion *m/z*	Retention Time (min)	Target Q1 Pre Bias (V)	Target Collision Energy (V)	Target Q3 Pre Bias (V)
26	PIPES (Internal standard)	Ion-pair RP/ESI negative	301.00	193.25	6.949	12	28	21
27	Sedoheptulose 7-phosphate	Ion-pair RP/ESI negative	288.90	97.10	7.001	23	23	17
28	Fructose 6-phosphate	Ion-pair RP/ESI negative	258.90	97.10	7.058	20	15	17
29	Ribose 5-phosphate	Ion-pair RP/ESI negative	229.10	96.95	7.067	18	13	18
30	Tryptophan	Ion-pair RP/ESI negative	203.10	116.15	7.070	16	18	21
31	a-Glycerophosphate	Ion-pair RP/ESI negative	171.10	79.10	7.226	13	16	13
32	Glutathione	Ion-pair RP/ESI negative	305.90	143.20	7.536	21	19	26
33	Ribulose 5-phosphate	Ion-pair RP/ESI negative	229.00	97.10	7.767	17	13	17
34	Orotate	Ion-pair RP/ESI negative	155.00	111.15	7.928	12	14	20
35	CMP	Ion-pair RP/ESI negative	322.00	79.10	8.099	25	28	13
36	Fructose 1-phosphate	Ion-pair RP/ESI negative	258.90	97.05	8.102	20	21	17
37	NAD	Ion-pair RP/ESI negative	662.10	540.10	8.281	26	18	26
38	Pyruvate	Ion-pair RP/ESI negative	87.00	43.05	8.318	10	11	14
39	DHAP	Ion-pair RP/ESI negative	168.90	97.05	8.608	13	12	17
40	UMP	Ion-pair RP/ESI negative	322.90	97.10	8.815	25	24	17
41	GMP	Ion-pair RP/ESI negative	362.00	79.10	8.995	29	28	13
42	Oxalacetate	Ion-pair RP/ESI negative	131.00	87.00	9.310	25	11	27
43	TMP	Ion-pair RP/ESI negative	321.00	79.10	9.719	25	38	14
44	AMP	Ion-pair RP/ESI negative	346.00	79.05	9.811	14	32	14
45	Nicotinate	Ion-pair RP/ESI negative	122.00	78.15	9.983	14	15	13

Table S5. *Cont.*

No	Metabolite	LC-MS Method */mode	Precursor ion *m/z*	Product ion *m/z*	Retention Time (min)	Target Q1 Pre Bias (V)	Target Collision Energy (V)	Target Q3 Pre Bias (V)
46	Pantothenate	Ion-pair RP/ESI negative	218.00	88.00	10.022	21	14	16
47	Succinate	Ion-pair RP/ESI negative	117.00	73.20	10.155	13	15	12
48	Fumarate	Ion-pair RP/ESI negative	115.00	71.10	10.278	13	10	12
49	cAMP	Ion-pair RP/ESI negative	328.00	134.10	10.465	15	27	24
50	Malate	Ion-pair RP/ESI negative	132.90	115.20	10.578	10	17	21
51	UDP-glucose	Ion-pair RP/ESI negative	564.80	323.10	10.712	24	26	15
52	2-Oxoglutarate	Ion-pair RP/ESI negative	145.00	101.20	10.745	11	13	18
53	CDP	Ion-pair RP/ESI negative	401.80	79.05	10.753	16	43	13
54	UDP	Ion-pair RP/ESI negative	402.90	79.05	10.807	16	48	13
55	NADP	Ion-pair RP/ESI negative	741.80	620.10	10.811	26	18	30
56	3Phosphoglycerate	Ion-pair RP/ESI negative	184.90	97.05	10.829	14	16	17
57	Fructose 2,6-bisphosphate	Ion-pair RP/ESI negative	338.90	241.15	10.834	26	19	27
58	Fructose 1,6-bisphosphate	Ion-pair RP/ESI negative	338.90	97.10	10.838	26	22	17
59	NADH	Ion-pair RP/ESI negative	664.00	78.95	10.876	24	57	13
60	Isocitrate	Ion-pair RP/ESI negative	190.90	73.20	10.891	13	22	26
61	Citrate	Ion-pair RP/ESI negative	190.90	87.00	10.892	13	18	14
62	ADP	Ion-pair RP/ESI negative	425.90	79.10	10.913	17	47	13
63	Bisphosphoglycerate	Ion-pair RP/ESI negative	265.00	167.15	10.919	11	18	29
64	Phosphoenolpyruvate	Ion-pair RP/ESI negative	167.00	78.95	10.928	15	13	13
65	2-Isopropylmalate	Ion-pair RP/ESI negative	175.00	115.20	10.998	13	16	21

Table S5. *Cont.*

No	Metabolite	LC-MS Method */mode	Precursor ion *m/z*	Product ion *m/z*	Retention Time (min)	Target Q1 Pre Bias (V)	Target Collision Energy (V)	Target Q3 Pre Bias (V)
66	FAD	Ion-pair RP/ESI negative	783.90	97.10	11.155	20	51	17
67	CTP	Ion-pair RP/ESI negative	481.90	159.10	11.171	19	36	29
68	GTP	Ion-pair RP/ESI negative	521.90	159.05	11.185	20	32	29
69	NADPH	Ion-pair RP/ESI negative	744.00	159.00	11.201	26	60	30
70	UTP	Ion-pair RP/ESI negative	482.90	159.10	11.206	19	36	29
71	ATP	Ion-pair RP/ESI negative	505.90	159.10	11.226	20	35	29
72	Acetyl-coA	Ion-pair RP/ESI negative	808.00	408.00	11.382	20	37	28
73	Cystine	RP/ESI positive	241.05	74.00	1.349	-25	-40	-14
74	Hydroxyproline	RP/ESI positive	131.70	85.95	1.437	-30	-19	-14
75	Cysteine	RP/ESI positive	122.00	59.00	1.482	-29	-40	-13
76	Homoserine	RP/ESI positive	119.70	74.15	1.514	-30	-15	-12
77	Alanine	RP/ESI positive	90.05	44.05	1.563	-16	-20	-19
78	Citrulline	RP/ESI positive	175.60	70.00	1.572	-30	-30	-30
79	Ornithine	RP/ESI positive	132.70	69.75	1.721	-30	-30	-30
80	Lysine	RP/ESI positive	146.70	83.95	1.783	-30	-25	-13
81	b-Alanine	RP/ESI positive	89.90	30.15	1.850	-14	-15	-30
82	Uracil	RP/ESI positive	113.15	70.05	2.278	-19	-43	-29
83	4-Aminobutyrate	RP/ESI positive	103.70	87.05	2.302	-30	-16	-15
84	Putrescine	RP/ESI positive	88.80	71.70	2.375	-30	-20	-30
85	Glycine	RP/ESI positive	118.05	43.05	2.829	-17	-40	-13
86	Valine	RP/ESI positive	118.10	72.10	2.857	-19	-10	-29
87	Spermidine	RP/ESI positive	145.70	72.20	3.130	-30	-20	-30
88	Hypoxanthine	RP/ESI positive	137.05	55.05	3.236	-21	-40	-28
89	Uridine	RP/ESI positive	244.90	113.05	3.322	-27	-10	-15
90	Guanine	RP/ESI positive	151.95	135.05	3.619	-29	-20	-16
91	S-Adenosylmethionine	RP/ESI positive	398.50	250.20	3.632	-30	-17	-25
92	Cytidine	RP/ESI positive	244.00	112.05	4.224	-16	-20	-16
93	Deoxycytidine	RP/ESI positive	228.10	112.10	5.629	-25	-10	-24
94	Leucine	RP/ESI positive	131.70	43.05	6.203	-30	-25	-17
95	Deoxyguanosine	RP/ESI positive	268.00	152.00	6.222	-18	-10	-15
96	Thymidine	RP/ESI positive	243.10	127.05	6.436	-27	-10	-29
97	Deoxyadenosine	RP/ESI positive	252.10	136.10	6.890	-17	-20	-27

*RP: reversed phase.
